# Genome sequence and genomic analysis of liver abscess caused by hypervirulent *Klebsiella pneumoniae*

**DOI:** 10.1007/s13205-023-03458-6

**Published:** 2023-02-03

**Authors:** Na Pei, Xin Liu, Zijuan Jian, Qun Yan, Qingxia Liu, Karsten Kristiansen, Junhua Li, Wenen Liu

**Affiliations:** 1 BGI-Shenzhen, Shenzhen, 518083 China; 2 Department of Clinical Laboratory, Xiangya Hospital, Central South University, Changsha, 410008 Hunan China; 3 Laboratory of Genomics and Molecular Biomedicine, Department of Biology, University of Copenhagen, Copenhagen, Denmark; 4 Shenzhen Key Laboratory of Unknown Pathogen Identification, Shenzhen, 518083 China

**Keywords:** *Klebsiella pneumoniae*, Liver abscess, Comparative genomics, Genome sequencing, Hypervirulent

## Abstract

Hypervirulent *Klebsiella pneumoniae* (hvKp) is an important pathotype with enhanced virulence features compared with classical *K. pneumoniae* (cKp). hvKp usually causes life-threatening infections in the community, often affecting young and healthy individuals. During the past few decades, hvKp-induced liver abscess has been increasingly reported in Asia and is emerging as a global disease. To better comprehend the molecular characteristics of hvKp-induced liver abscess and recognize the global dissemination of hypervirulent strains with resistance determinants, we sequenced the whole genome of 26 K*. pneumoniae* strains from patients with liver abscess (KLA) and investigated the clinical factors related to different phenotype groups. The epidemiology, virulence-related factors, and antimicrobial resistance determinants were also discussed. The age, gender, and whether being hospitalized showed no differences among the string-positive and -negative groups were also studied. The assembly and annotation suggested that most of the 26 new liver abscess-causing hvKp strains were ST23-K1 or ST86-K2, and only one of the strains exhibited multidrug resistance. Compared with the existing 36 global liver abscess genome sequences, higher sequence type and virulence gene diversity were found in the new genomes. The clinical characteristics and genomic data of the isolated strains will enrich our knowledge for comparative genomic studies, allowing the better understanding of hvKp characteristics and evolution.

**Electronic supplementary material:**

The online version of this article (10.1007/s13205-023-03458-6) contains supplementary material, which is available to authorized users.

## Introduction

Liver abscess caused by *Klebsiella pneumoniae* is an invasive disease emerging as a global disease. The incidence of pyogenic liver abscess has remarkably increased from 10.83 to 15.45 per 100,000 person-years in the past decade (Chen et al. 2016). *K. pneumoniae* is the predominant pathogen causing liver abscess, and nearly 91% of these liver abscess-causing *K. pneumoniae* (KLA) strains were hypervirulent(Jun 2018). This KLA-caused invasive disease was first described in 1986 in Taiwan (Liu et al. 1986). HvKp is a variant pathotype of *K. pneumoniae* that demonstrates increased virulence with a propensity to cause liver abscess relative to cKp. KLA has unique phenotypic and genotypic characteristics. Subsequently, KLA was reported in many southeast Asian countries and has become a significant health concern in Asia. HvKp has recently been increasingly recognized in North America, Europe, and Australia which poses a huge challenge to global public health (Siu et al. 2012).

To date, the virulence, antibiotic resistance determinants, and the global spread of hvKp isolates from liver abscesses have not been fully characterized. The hypermucoviscosity (HV) phenotype can be used as an approximate marker for isolates from KLA patients. However, in recent years, many KLA strains have been reported without their HV phenotypes. Whole genome sequencing (WGS) can be used for studying the epidemiology of pathogens such as *K. pneumoniae* and for their surveillance. It also allows the study of the high virulence mechanism and provides more information about the evolution and geographical spread of clinical strains (Wyres et al. 2020). In this study, we sequenced and analyzed the whole genomes of 26 new isolates from KLA patients and compared them with those of strains previously reported from other parts of the world, hoping to expand the understanding of genetic determinants of hvKp.

## Materials and methods

### Clinical strains and phenotypic characterization

Twenty-six liver abscess-causing hvKp isolates were collected and cultured from the puncture fluids of clinically confirmed patients between May 24, 2013 and June 14, 2018 in Xiangya Hospital Central South University. This is a tertiary hospital in Hunan Province, Changsha, China. All isolates were identified by MALDI-TOF MS, and the minimum inhibitory concentration (MIC) was determined using the Vitek compact 2 system (bio Mérieux, Marcy l'Étoile, France). The CLSI document M100-S15 was used to interpret MIC. The present study was approved by the Human Ethics Committee of Xiangya Hospital of Central South University (No. 201806861).

We used the string test to identify the mucoid phenotype as previously described (Shon and Russo 2012). When an inoculation loop can generate a > 5-mm-long viscous string from colonies of a KLA strain, this strain was regarded as positive; otherwise, it was considered negative. A picture of the mucoid phenotype of isolates from KLA patients is shown in Supplementary Fig. S2.

### Sequencing and genome assembly

The *K. pneumoniae* isolates were grown overnight in LB broth at 37 ℃, and total DNA was extracted from the harvested cells and centrifuged at 10,000 rpm for 1 min. The supernatants were discarded, and the pellets were extracted using the TIANamp Bacteria DNA Kit (TIANGEN BIOTECH (Beijing) CO, LTD) according to the manufacturer’s instructions. The DNA was subjected to paired-end WGS on the BGISEQ-500 sequencing system (MGI, Shenzhen, China, pair-end 150 bp).

The sequencing reads were quality controlled using Fastp (parameter: − q 20; − l 30) (Chen et al. 2018) and SOAPnuke (parameter: − Q 2) (Chen, et al. 2018). The reads with a quality lower than Q20 and length of < 30 were removed prior to assembly. The clean reads were assembled in SPAdes (parameter: − careful; − sc) (Prjibelski et al. 2020) using k-mer sizes of 55, 77, and 99. Assemblies with a genome size of 5.0–7.0 Mb or a GC content of 40–60% were retained. Then, the assemblies were polished using all trimmed reads with bwa (parameter: index; mem) (Li and Durbin 2009), SAMTOOLS (parameter: view -bS; sort) (Li et al. 2009), and PILON (parameter: − fix all; − changes) (Walker et al. 2014).

### Genome annotation and analysis

The in silico multilocus sequence typing (MLST) of each genome was performed using mlst (parameter: default) according to the PubMLST database. Kaptive (parameter: default) (Wyres et al. 2016) was used to identify the K-locus of the whole genome data.

Prokka (parameter: − kingdom bacteria; − species pneumoniae; − evalue 1e-06) (Seemann 2014) was used to annotate the de novo assemblies with predicted genes. The output of the GFF3 format was used as an input for Roary v3.12.0 (Page et al. 2015), choosing a minimum blastp identity of 95, and genes present in 90% of the isolates were defined as core genes. The database of virulence genes in *K. pneumoniae* was downloaded from BIGSdb (http://bigsdb.Pasteur.fr/klebsiella/klebsiella.html). The virulence genes were predicted using Kleborate (parameter: default) (Lam et al. 2021) and BLAST search against the database with 95% coverage and 90% identity. Using ResFinder (parameter: − min_cov 0.6; − threshold 0.8) (Zankari et al. 2012), drug resistance genes of hvKp genomes were predicted.

### Single nucleotide polymorphism calling and phylogenetic analysis

The genomic sequences of 26 samples were compared with a global collection of samples of *K. pneumoniae*-induced liver abscess. Previously reported 36 global sequencing reads of hvKp isolates were downloaded from NCBI (https://www.ncbi.nlm.nih.gov) and ENA (https://www.ebi.ac.uk/ena) (Supplementary Table S1) for comparison (Struve et al. 2015; Lee et al. 2016). The downloaded reads were subsequently qualified using the aforementioned QC method.

SNPs were identified by aligning the reads from each isolate to a reference genome (*K. pneumoniae* strain NTUH-K2044, accession number: NC_006625.1) using Snippy (https://github.com/tseemann/snippy). The snippy-core was used to produce an alignment of core SNPs of all genomes. Recombination events in the core genome alignment were assessed and removed using Gubbins (parameter: default) (Croucher et al. 2015). With SNP sites, SNPs were extracted from the core SNP alignment after removing recombinations. IQtree (parameter: − m MFP; − T AUTO) (Minh et al. 2020) was applied to construct a maximum likelihood (ML) tree. iTOL (Letunic and Bork 2019) was used for visualizing the phylogenetic tree.

## Results and discussion

### Clinical characteristics of the 26 isolates from KLA patients

In total, 26 isolates were collected from patients with liver abscess between May 2013 and June 2018. Among them, 53.8% of the patients were men in the string-positive group. The majority of the patients were aged > 40 years (age range 27–73 years, median age 53 years). Sixteen patients were hospitalized. The majority of isolates originated from drainage fluids of KLA patients (*n* = 22) and four blood samples were also retained. Most patients were from the surgery department (*n* = 11), followed by the infectious diseases department (*n* = 10). No significant differences were noted between the groups (Table 1).

**Table 1:** Clinical characteristics of 26 patients infected with liver abscess-causing *K. pneumoniae*

	String positive^a^ (*N* = 13)	String negative (*N* = 13)	*P* value
Age			
≤ 40	1 (7.7%)	1 (7.7%)	0.664
40–50	5 (38.5%)	2 (15.4%)	
51–60	4 (30.8%)	5 (38.5%)	
≥ 60	3 (23.1%)	5 (38.5%)	
Gender			
Male	7 (53.8%)	6 (46.2%)	1
Female	6 (46.2%)	7 (53.8%)	
Hospitalized^b^			
Yes	6 (46.2%)	10 (76.9%)	0.39
No	5 (38.5%)	3 (23.1%)	
ND	2 (15.4%)	0 (0%)	
Sample type			
Blood	1 (7.7%)	3 (23.1%)	0.593
KLA drainage fluid	12 (92.3%)	10(76.9%)	
Department			
Outpatient	1 (7.7%)	1 (7.7%)	0.447
Medicine	1 (7.7%)	2 (15.4%)	
Surgery	4 (30.8%)	7 (53.8%)	
Infectious diseases	7 (53.8%)	3 (23.1%)	

### Genome assembly and annotation overview

The assembly results and integrity of the new genomes were evaluated in detail. The genome assembly statistics showed that our assembly lengths were between 5.1 and 5.6 MB, and the number of contigs in the sequences were 50–149 (Table 2). Benchmarking Universal Single-Copy Orthologs (Simao et al. 2015) were used to estimate genome completeness. The results showed that our assemblies have a high completeness (> 98.4%) (Fig. S1). In pan-genomic analysis, according to gene prevalence within the isolates, 8868 gene families were classified as core (genes present in 90–100% of the genomes) and accessory genomes (genes present in < 90% of the genomes). In total, 4269 core genes (48.1%) were identified in the 26 new KLA genomes.

**Table 2:** Genome overview of 26 isolates from KLA patients

	Total length^a^	GC^b^ (%)	N50^c^	Contigs^d^	CDS^e^
KP0001	5,480,069	57.18	359,721	78	5077
KP0002	5,480,541	57.37	332,195	75	5030
KP0003	5,423,848	57.3	363,472	82	5016
KP0004	5,606,836	57.23	441,157	79	5143
KP0005	5,622,848	57.23	381,135	149	5130
KP0006	5,603,731	57.19	381,063	134	5130
KP0007	5,655,621	57.18	481,343	102	5203
KP0008	5,634,535	57.18	369,490	73	5182
KP0009	5,529,534	57.19	369,946	79	5047
KP0010	5,647,901	57.18	397,260	88	5188
KP0011	5,499,464	57.34	333,976	74	5039
KP0012	5,530,616	57.31	374,873	69	5105
KP0013	5,412,723	57.25	376,646	58	5031
KP0014	5,351,089	57.43	380,357	50	4923
KP0015	5,287,954	57.5	373,147	87	4933
KP0016	5,213,123	57.61	349,841	87	4817
KP0017	5,466,539	57.05	330,217	62	4991
KP0018	5,195,785	57.51	400,945	80	4787
KP0019	5,183,701	57.63	460,484	50	4789
KP0020	5,170,661	57.66	376,114	53	4793
KP0021	5,538,432	57.31	385,247	51	5159
KP0022	5,421,931	57.27	330,055	73	5038
KP0023	5,472,424	57.24	356,656	62	5054
KP0024	5,423,850	57.27	330,080	81	5041
KP0025	5,419,785	57.27	330,022	65	5038
KP0026	5,439,121	57.26	371,946	66	5007

To evaluate the factors for distinguishing the different strains, which may lead to phenotypic differences, we thoroughly investigated the accessory genes. Of the 4599 accessory genes, most were annotated as hypothetical proteins (*n* = 3118, 68%). Of the remaining 1481 genes, 516 genes (35%) were strain-specific, 965 genes were found in at least 2 strains, and 101 genes were found in more than 20 strains. Some interesting genes have caught our attention, for example, 6 beta-lactamase resistance-related genes in KP0003, KP0014, and KP0017; 2 CRISPER system-related genes in 11 strains, 14 genes for multi-drug resistance proteins, 11 genes for fimbria, and 10 genes for the Type IV secretion system in at least 1 strain (Supplementary Table S2).

^a^String positive is defined as the viscous string > 5 mm in length. Values in the table are reported as the number (%) of patients unless otherwise indicated

^b^Hospitalized: yes: inpatient; no: outpatient; ND: data not available

^a^The total length of the Kp genome

^b^The GC content

^c^N50 is defined as the sequence length of the shortest contig at 50% of the total genome length

^d^Contig is a set of overlapping DNA segments that together represent a consensus region of DNA

^e^CDS: Coding sequence

### Virulence and drug resistance determinants of isolates from patients with liver abscesses

**Fig. 1:**
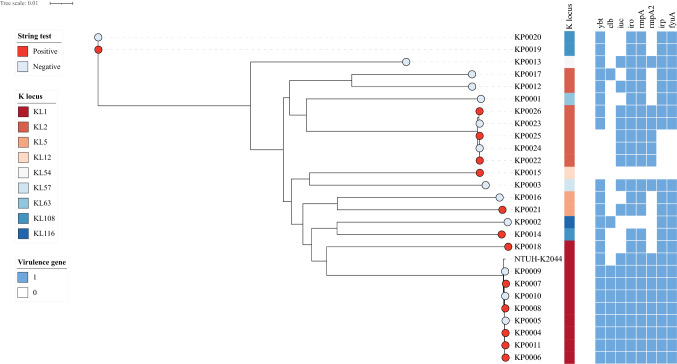
Core SNP tree of 26 new isolates from patients with liver abscesses. The red color of the isolate tips represent the positive string test results, and metadata columns show the virulence genes and K-locus types. The tree was rooted with the outgroup *K. pneumoniae* strain NTUH-K2044. The scale bar indicates the number of substitutions per site

We attempted to access the virulence factors contributing to these new isolates from KLA patients, the distribution of main virulence genes, and the K-locus in *K. pneumoniae,* as shown in Fig. 1. The K-locus is 10–30 kbp in length and codes for the capsule synthesis process of *K. pneumoniae. rmpA*, which activates capsule production, was detected in 92.3% (*n* = 24) of the 26 isolates. *ybt* encoding for the yersiniabactin system was detected in 84.6% (*n* = 22) of the isolates. The receptor gene *fyuA* (Hancock et al. 2008) and biosynthesis gene *irp* (Pelludat et al. 1998) were detected in the same proportion as *ybt* in all isolates. Regarding other siderophore systems, *iuc* encoding for the aerobactin system and *iro* encoding for the salmochelin system were identified in 65.4% (*n* = 17) and 92.3% (*n* = 24) of the isolates from KLA patients, respectively. *clb* encoding the genotoxic polyketide colibactin, which was recently found to contribute to colorectal cancer(Strakova et al. 2021), was found in 38.5% of the isolates (*n* = 10). The prevalence rates of *ybt*, *irp* and *fyuA* in the four blood isolates were all 100%, but all showed the absence of *iuc* gene.

Nine different K loci were identified in the 26 genomes, and the most common K loci were KL1 (*n* = 9) and KL2 (*n* = 7), which account for 61.5% of *K. pneumoniae* isolates. While ST23 was the dominant sequence type in the KL1 isolates (8/9) and had the same virulence determinants. In contrast, in the KL2 serotype, 71.4% (5/7) of the strains were ST86 and the remaining strains included 1 ST25 and 1 ST65. Among these strains, ST23 and ST86 have been reported to be the most common hvKp-associated clones (Choby et al. 2020). ST23 was the main sequence type in isolates from KLA patients and was strongly associated with the K1 capsular serotype (*p* < 0.001), which is often detected among hvKp in different investigations and collections (Wyres et al. 2020). In addition to these two serotypes, K5, K12, K54, K57, K63, K108, and K116 capsular serotypes were detected in the genomes. Among these serotypes, K5, K54, K57 (Liu et al. 2014), K63 (Lee et al. 2017), and K108 (Lan et al. 2021) were found to be related to hvKp in previous studies, while K12 and K116 were first reported in the present study. Approximately 50% isolates (*n* = 13) were string-test positive with their K loci distributed as follows: 6 KL1, 3 KL2, 1 KL5, 1 KL12, and 2 KL108. Despite the absence of *rmpA* in KP0015, the HV test was still positive, which suggests that *rmpA* is not required for string test positivity. No association was observed between other virulence genes and the string-test positive phenotype.

To investigate whether a co-occurrence of antimicrobial resistance (AMR) and virulence genes existed in the isolates from KLA patients, AMR genes were also analyzed among these 26 genomes. Multiple AMR genes associated with resistance to aminoglycoside, β-lactam, fosfomycin, phenicol, quinolone, rifampicin, sulphonamide, tetracycline, and trimethoprim antibiotics were identified (Table S3). All strains contained the efflux pump oxqA/B gene, which was the core gene conferring quinolone resistance (Kiaei et al. 2019). All strains were ampicillin-resistant, and most strains (*n* = 20) exhibited complete or intermediate resistance to nitrofurantoin. Combining the results of the AST test and the prediction of drug resistance genes, we found that no strain showed multidrug resistance except strain KP0015, which showed intermediate or complete resistance to nine drugs and carried multiple *bla* genes including *bla*_SHV-81_, *bla*_CTX-M-3_, and *bla*_TEM-1B_, thus exhibiting extensive drug resistance.

### The phylogenetic tree of KLA strain collections

**Fig. 2:**
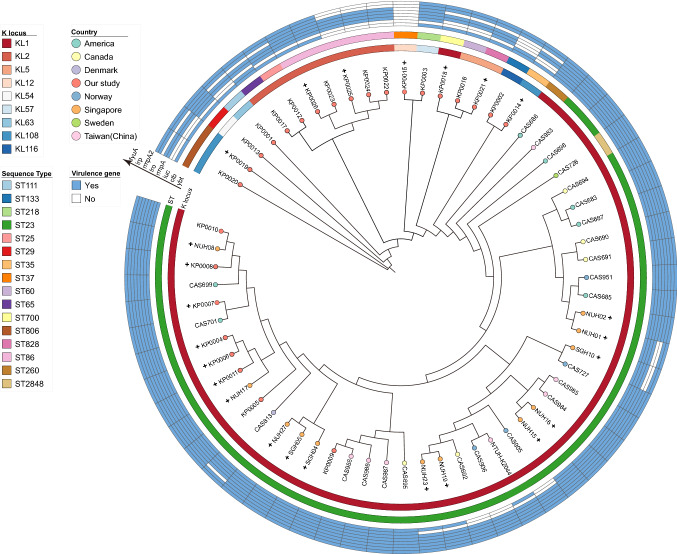
Phylogenetic tree based on core SNPs from 62 new and global isolates from patients with liver abscesses. K loci are shown in the inner ring and labeled. STs are colored in the middle ring. In the outer ring, virulence genes are labeled blue and white means none. The colors of the isolate tips represent the country of isolation, and the isolate name with a plus sign in front indicates that the string test result is positive

Until now, many clinical studies have investigated about KLA, but very few whole genome sequences were available. Therefore, we downloaded almost all KLA sequences available in the public database to date from 1996 to 2012. A core SNPs tree was constructed to provide the high-resolution phylogenetic structure of the 26 new and 36 publicly available isolates from KLA patients. Based on the phylogenetic structure, all the 62 isolates were categorized into two major lineages (Fig. 2). Significant differences were observed in the distribution of the accessory genes and the sequence types between the two lineages. One lineage (lineage 1) contained most KLA and ST23 strains and nearly all the common virulence genes, while the other lineage (lineage 2) showed diversity in STs and the number of virulent genes. The annotated virulent genes were also fewer than that in lineage 1. Lineage 1 can also be classified into two sublineages: one containing 8 new isolates from KLA patients (sublineage1) and the other containing all public genomes (sublineage2). All ST23-K1 strains (*n* = 45, 72.6%) were mutated and clustered in lineage 1 compared with the reference strain NTUH-K2044 including 8 new isolates from KLA patients. Our new ST23-K1 isolates were more clustered in sublineage1 closely related to the strains in Singapore, America, and Denmark; most of them (*n* = 5, 62.5%) were string-test positive. In addition, lineage 1 showed different regional distributions, suggesting that most isolates from KLA patients are from parts of Asia, with sporadic cases reported from Europe and America. Lineage 2 consisted of 18 new isolates from KLA patients and showed great sequence diversity. The most common sequence type was ST86 (*n* = 5), and four strains had a deletion of *ybt*; 38.9% (*n* = 7) isolates were string-test positive. Further studies are required to understand the reason for this difference and its effect on the KLA phenotype.

## Conclusions

Invasive liver abscess caused by *K. pneumoniae* has become a major health concern worldwide, especially in the Asia Pacific region. Although some epidemiological studies have reported on *K. pneumoniae*-induced invasive liver abscess, limited high-quality genomes of the infecting strains can be found in the database. In the present study, we sequenced the whole genomes of 26 isolates from KLA patients and investigated about clinical factors. Further, we compared these genomes with those of 36 isolates from KLA patients previously reported from other parts of the globe, hoping to expand the understanding of evolution, virulence, and resistance factors of the strains from KLA patients.

## Electronic supplementary material

Below is the link to the electronic supplementary material.

Supplementary material 1 (docx 680 kb)

Supplementary material 2 (2,738 kb)

## Data Availability

The sequence reads of the new 26 genomes ranged from 370 to 1200 Mb with a mean depth of 86 × . The genome sequences were available in GenBank with the accession numbers JAHTJI000000000 to JAHTKH000000000, and the CNGB Nucleotide Sequence Archive CNP0002056.

## Code availability

No new code was generated in this study. Softwares used for data processing and analysis were stored in public repository, with the versions and parameters described in the Methods section of the manuscript. Default parameters were used if not declared.
