# Supplementary material for: Genome sequence and genomic analysis of liver abscess caused by hypervirulent Klebsiella pneumoniae
Source: 3 Biotech. 2023 Feb 3;13(3):76. doi: 10.1007/s13205-023-03458-6 (PMC9898476; doi:10.1007/s13205-023-03458-6)
Supplement: Supplementary file 1 — Supplementary material 1 (docx 680 kb) [file 13205_2023_3458_MOESM1_ESM.docx]

**Supplementary information**

**Figure S1**. **Completeness assessments of the KLA genome**


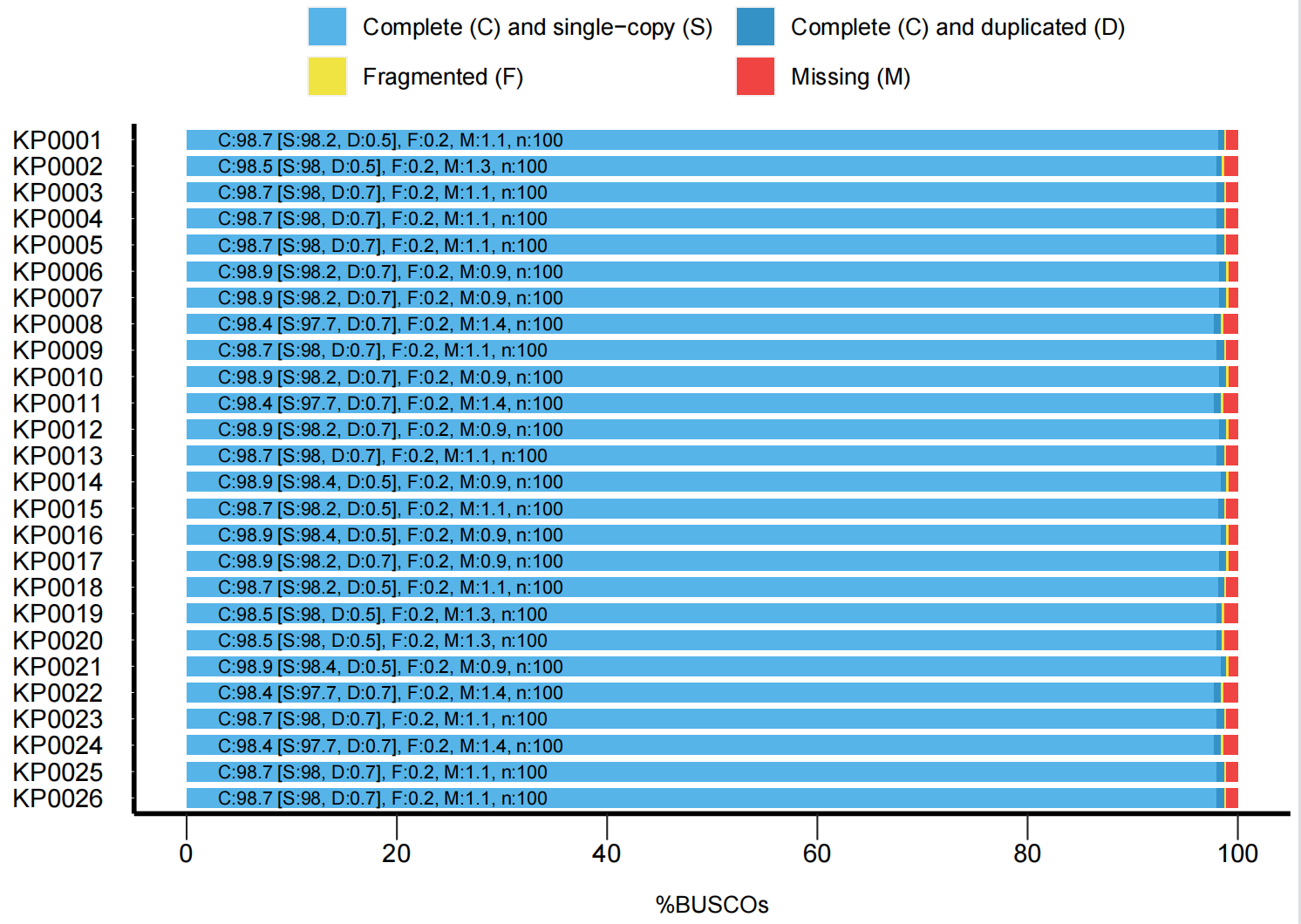


Bar charts produced with the BUSCO plotting tool show proportions classified as and single-copy(blues), complete and duplicated (dark blue), fragmented (yellow), and missing (red).

**Figure S2** **Picture of mucoid phenotype of KLA strains**


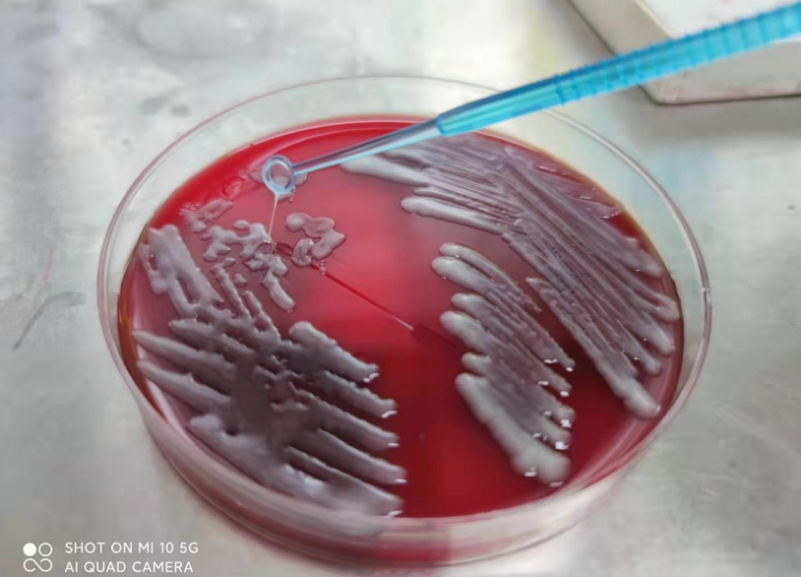


**Table S1 Public available 36 KLA genome sequences to date**

**Table S2 Accessory gene annotation of 26 new KLA strains**

**Table S3. Antibiotic resistance characteristics and AMR genes of 26 new isolates.**
